# The role of radiofrequency therapy in the treatment of Morton's neuroma: A case report

**DOI:** 10.1016/j.ijscr.2025.111424

**Published:** 2025-05-13

**Authors:** Romy Deviandri, Raymond Santoso, Nasywa Devina Mecca, Najmi Khairussyifa, Kayla Annasya, Rima Farahdina, Muhammad Wiranata

**Affiliations:** a Department of Surgery, Faculty of Medicine, Universitas Riau, Arifin Achmad Hospital, Pekanbaru, Indonesia; b Department of Orthopedics, University of Groningen, University Medical Center Groningen, Groningen, the Netherlands; c Department of Orthopedics, Faculty of Medicine, Universitas Padjajaran, Hasan Sadikin Hospital, Bandung, Indonesia

**Keywords:** Neuroma, Radiofrequency therapies, Questionnaire, Case report

## Abstract

**Introduction and importance:**

Morton's Neuroma is a neuropathy of the forefoot, specifically located on the interdigital nerve, and is associated with the thickening of the intermetatarsal ligament and nerve fibrotic. The management of Morton's Neuroma is challenging. Various treatments have been introduced for treating this lesion, starting from conservative treatment using the metatarsal pad, physical therapy, and corticosteroid injection to surgical treatment. This report highlights a novel approach to Morton's Neuroma using a pulsed radiofrequency (PRF).

**Case presentation:**

A 53-year-old female, working as a tailor, was transferred to the orthopedic clinic after complaining of pain in her right plantar surface, and she was restricted from standing, walking, and descending stairs. The patient was administered analgesics and anti-inflammatory drugs, but the pain worsened. In the physical examination, there are no signs of trauma on the right foot. There was tenderness in the 2nd and 3rd metatarsal space, and increasing pain was obvious when the foot was squeezed. Magnetic Resonance Imaging (MRI) represents Neuroma in the intermetatarsal II-III segment. After a thorough examination, the patient was diagnosed with Morton Neuroma.

**Clinical discussion:**

We performed a pulsed radio frequency (PRF) with linear Ultrasonography (USG) guidance from the plantar of the foot. PRF delivers in two cycles, each lasting 4 min. The patient was followed for six months. The Visual Analog Scale (VAS) and Foot and Ankle Disability Index (FADI) score were used to evaluate the patient's outcome.

**Conclusion:**

A PRF approach is a favorable option for treating Morton's neuroma patients. VAS and FADI scores represent good functional outcomes.

## Highlights

• Morton Neuroma is one of the rare condition which affect interdigital nerve.
• Performing a minimal invasive approach may improve patient's quality of life.
• Pulsed Radiofrequency was chosen to minimize progressed pain of foot.
• Foot and Ankle Disability Index and Visual Analog Scale indicate favorable results.

## 1 Introduction

Morton's Neuroma is a painful lesion of the interdigital nerve associated with perineural fibrosis and nerve thickening, commonly seen in middle-aged women [1]. The incidence is 87.5 per 100,000 persons [2]. Rest pain was reported in 25 % population [3]. This condition can restrict weight-bearing activities and impacting the quality of life [4]. The treatment goal is to reduce pain and initial management includes orthotics, physical therapy, and corticosteroids. In severe cases, surgery may be necessary [4,5].

Pulsed Radiofrequency (PRF) is an emerging treatment for pain management with promising results due to its minimally invasive approach. This method delivers short energy in a short period, divided into several cycles. It is safe and easier to perform [5]. To lessen pain signals from specific nerve tissue, the electric produced by radio waves is employed to heat that area. Chronic knee pain has been treated with PRF, which includes thermal and cooled radiofrequency [5]. PRF is a particular radiofrequency in that relatively no tissue damage occurs as the temperature is set at a limit of 42 °C. This study aimed to describe the treatment of Morton's Neuroma and reported using SCARE Guideline 2023 [6]. Patients' outcomes were measured with the Foot and Ankle Disability Index (FADI) and Visual Analog Scale (VAS).

## 2 Case presentation

A 53-year-old female working as a tailor went to the clinic after complaining of pain in her right plantar surface and right knee 6 months before clinic visitation. The patient described the pain as stabbing with an electrical sensation and got worse each day. The patient was administered analgesics and anti-inflammatory drugs, but the pain worsened. Next, the patient was advised to use external support, but the symptoms were not relieved. Then, the patient was transferred to the orthopedic clinic for further examination. There was no history of trauma, hospitalization, or long-term medication use, and she has never smoked in her entire life. Her family has no history of illness.

The vital signs remained stable, and the Body Mass Index (BMI) showed overweight. There was no sign of deformities while performing a physical examination on the right plantar surface. However, there was tenderness in the 2nd and 3rd metatarsal space, and increasing pain was obvious when the foot was squeezed. The patient was restricted from performing dynamic activities, such as standing, walking, squatting, and going up and down stairs. The FADI measurement tool was conducted to observe the patient's ability to move her foot and ankle, and it scored 50, with a VAS score of 7. There was no sign of neurological deficit in the patient. A particular blood test, routine blood test, erythrocyte sedimentation rate (ESR), and C-reactive protein (CRP) revealed good results, with no sign of infection.

**Fig. 1:**
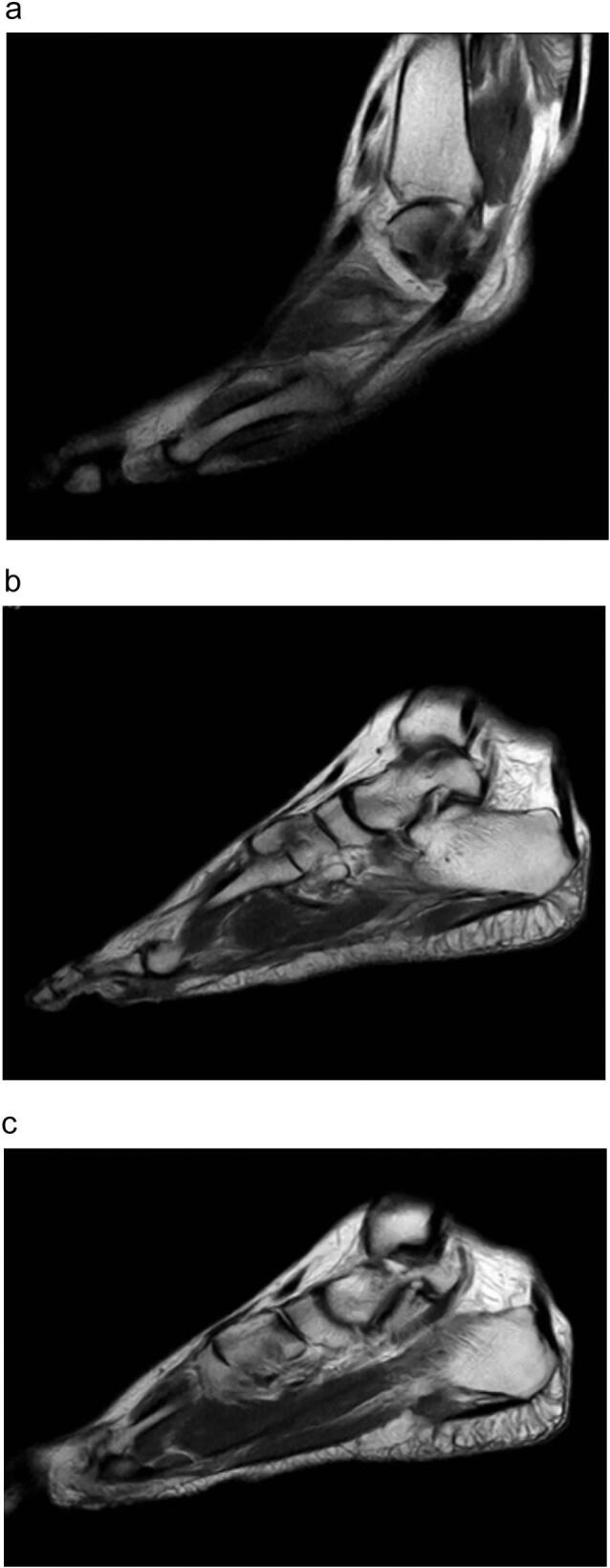
MRI of Morton's Neuroma on the right foot shows thickening of nerve cells on the intermetatarsal space 2nd-3rd.

A conventional radiology examination shows no sign of fracture. A further investigation with MRI was performed and revealed a thickening of nerve cells on the distal intrametatarsal 2–3, called Neuroma (Fig. 1).

Our treatment aimed to minimize nerve pain and improve the patient's quality of life. PRF treatment was chosen to overcome the patient's problem. Moreover, a minimally invasive approach may improve patient mobilization. The patient has no comorbidities that would affect our treatment plan. Informed consent was obtained. Our hospital's orthopedic surgeon and pain interventionist performed the procedure.

The intervention instruments, such as a linear Ultrasonography (USG, Wisonic Navi Color Doppler; China), C-Arm Fluoroscopy, and the PRF kit, were prepared before the procedure. The PRF kit consists of a cannula, PRF probe, and generator to generate heat (Cosman RFG 4, United States).

**Fig. 2:**
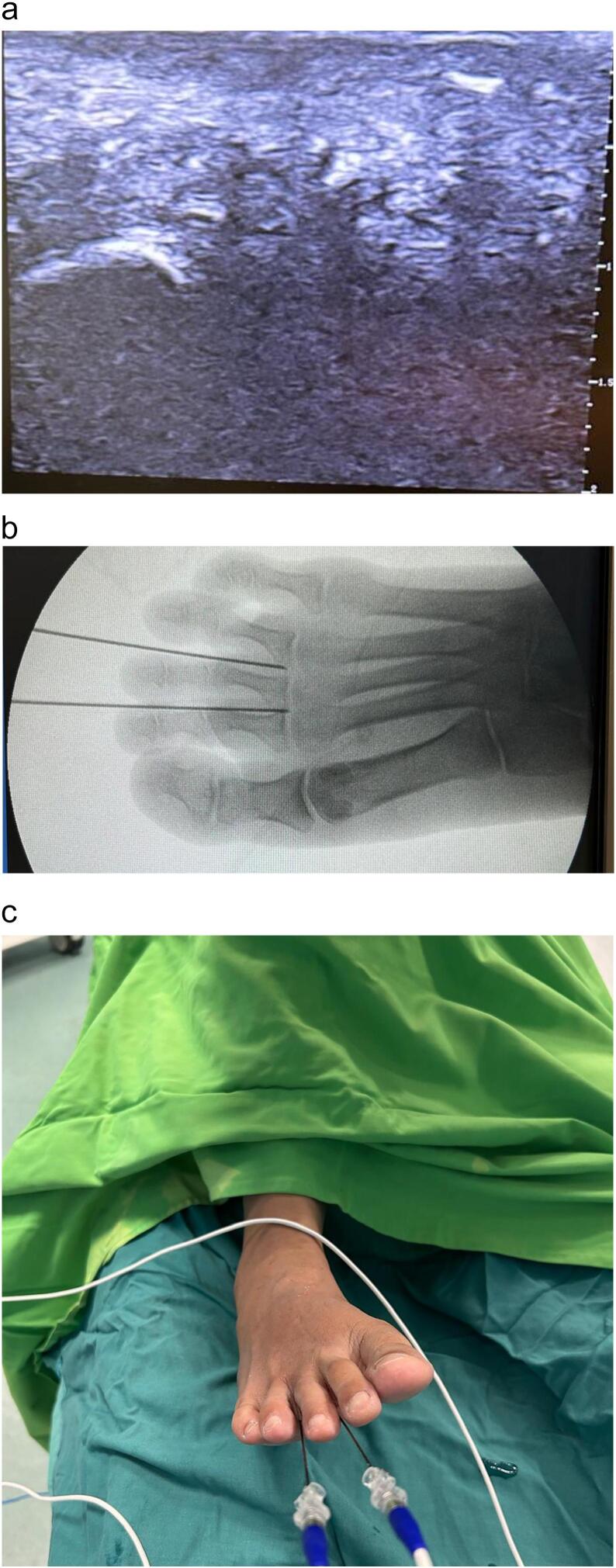
A linear ultrasound shows a hypoechoic Morton's Neuroma **(a).** A cannula position at Morton's Neuroma lesion around the distal part of two-thirds of the intermetatarsal was confirmed by fluoroscopy **(b).** Two RF cannulas were inserted at the foot's 2nd and 3rd web space for transmitting PRF **(c).**

The patient is in a supine position with local anesthesia applied around the affected area. We used a linear USG starting from the plantar side of the foot during treatment to locate the lesion and guide the thin cannula insertion. The cannula is inserted between the toes, targeting intermetatarsal neuroma sites. Next, the C-Arm Fluoroscopy is also used to confirm the location. The PRF probe is connected to the generator, producing short radiofrequency energy to generate heat at lower temperatures at the probe's tip. This procedure begins in two cycles, each lasting 4 min, reaching 42 °C of heat (Fig. 2).

After the procedure, the patient's hemodynamics remained stable, and no significant complications were found. The patient was instructed to prevent heavy activities. As pain was tolerated, mobilization was permitted. One day after treatment, the patient's VAS score was 3, which indicates mild pain, but a discoloration of the injection sites was still found. Besides, the FADI showed a score of 75.

Two weeks after treatment, the patient came to the orthopedic outpatient clinic to follow up and implement certain examinations. The patient was instructed to perform standing, walking, and going up and down stairs. The patient was trained to perform several FADI examinations and showed improvement. The VAS score was decreased to 2, and the FADI scored 80.

The patient returned to an orthopedic surgeon one month after treatment and showed better results. The VAS score was decreased to 1, and the FADI showed a score of 90, which points out an improvement. The patient's perspective shows improvement after surgery; she was able to perform daily activities after treatment.

## 3 Discussion

Morton's Neuroma is a condition where the intermetatarsal ligament and nerve branches are repeatedly compressed due to the thickening of the intermetatarsal nerve tissue, the most common site is between the third and fourth toes [7]. The repeated compression leads to discomfort, swelling, and sharp and burning pain in the foot, especially on the plantar surface. Commonly, the symptoms worsen when doing high-impact activities such as running or wearing tight footwear [8]. Based on our report, the patient felt tingling with an electrical sensation on the right foot's plantar surface during daily activities.

RF has been used as a therapeutic method for managing peripheral nerve pain; the duration of action is better, and the risk of tissue injury is minimal. RF is one of the most popular approaches; the mechanism points out neuromodulation with minimal thermal disruption on the surrounding tissue. RF includes conventional (thermal), cooled, and PRF [5]. PRF is a particular RF in that relatively no tissue damage occurs as the temperature is set at a limit of 42 °C. On the other hand, PRF is often used in relatively low thermal ablation, with results revealing moderate nerve structure disruption. PRF inhibits un-myelinated C-fibers while exposed to a needle tip on the electromagnetic surface [9]. Subsequently, PRF will disrupt the nociceptors segment of nerve fibers, and slowly, the nerve will regenerate its complex integrity.

Initial management of Morton's Neuroma includes orthotics, physical therapy, and corticosteroids for pain reduction. An alternative treatment for managing Morton's Neuroma, known as neuroma pad or metatarsal pad, can be an option. This conservative modality may reduce mechanical compression of the interdigital nerve [10]. In severe cases, surgery may be necessary, though it carries a high risk of nerve damage. However, the recurrence of Morton's Neuroma remains a significant concern in its management. Recurrence rates are reported to range between 5 % and 30 %, often attributed to incomplete resection, nerve regeneration, or the formation of stump neuroma [11,12]. Stump neuroma in recurrent interdigital neuromas is primarily attributed to aberrant nerve regeneration after neurectomy. It is thought to result from plantar-directed nerve branches tethering the digital nerve, preventing its proximal retraction during resection. This leaves the transected nerve end in the webspace, promoting disorganized regeneration and neuroma formation. Addressing these tethering branches during surgery is crucial to reduce this risk [13,14].

Based on our report, we implement PRF to manage Morton's Neuroma on the intermetatarsal II - III, specifically at the interdigital nerve of the affected site. PRF was chosen due to its safe and better mechanism of action. Some limitations of this PRF approach, including variable and total energy transfer when delivering this treatment, were still not always certain. However, PRF is preferable to conventional thermal RF because of the lower heat energy produced in a cycle, reducing the risk of local burn injury in the affected site. Furthermore, this treatment is cost-effective because our institution's technical equipment is affordable. A certain limitation still found, including variable and total energy transfer, was not always known [9]. We prefer PRF as our treatment modality because this method is a minimally invasive approach that lowers complications, considering a cost-effective treatment with a promising outcome.

The Foot and Ankle Disability Index (FADI) was used to assess the patient's functional outcome. The FADI is an assessment tool used to evaluate patient outcomes, focusing on foot and ankle area, by comparing patient's scores before and after receiving treatment, particularly in individuals with musculoskeletal disorders, injuries, or gait abnormalities, typically includes measurements of Range of Motion (ROM), strength, balance, and pain tolerance, helping clinicians determine functional impairment and monitor rehabilitation progress [ 15]. The FADI assesses the patient through daily activity and pain tolerance score, where each item has its score. The total score of FADI is 104, indicating good function and good outcomes; the lower the patient gets, the worse the patient's outcomes [ 13,15]. In this case, the improvement in the FADI score remains stable and promising. The FADI score before treatment started was 50, which showed a limitation for the patient. But, two weeks after the intervention, the score went up to 80, which showed an improvement from the treatment. Subsequently, six months after treatment, the score went up to 96, indicating good improvement and promising results. However, further observation must be conducted periodically to maintain the results after treatment. Considering the risk of recurrence regarding Morton's Neuroma, the observation needs an extended period using proper measurement tools to secure the quality of the treatment [16].

## 4 Conclusion

Morton's neuroma treatment is challenging. Treating Morton's Neuroma by PRF could be preferable to others because of this minimally invasive method with good outcomes.

## Author's contribution

Study concept or design, data analysis or interpretation: Romy Deviandri

Data Collector and writter: Raymond Santoso^3^, Najmi Khairussyifa^1^, Kayla Annasya^1^,Rima Farahdina^1^, Muhammad Wiranata^1^

## Consent

The patient provided written informed consent to publish this case report and its images. A copy of the consent form is available for review by the Editor-in-Chief upon request.

## Ethical approval

This case report did not need ethical approval from our institution.

## Guarantor

Romy Deviandri

## Research registration number

In this study, the reported case was not a “First in man” studies.

## Sourcing of funds

No specific funding from public, commercial, or non-profit organizations was found to fund contributions for this case report.

## Declaration of competing interest

The authors have no conflicts of interest regarding the case.
